# Research on Psychological Resilience, Digital Competence, and Self-Efficacy in Online TCFL Teachers

**DOI:** 10.3390/bs15030366

**Published:** 2025-03-14

**Authors:** Qian Shi, Xiangdong Xu, Youwen Zhang, Bo Hu

**Affiliations:** 1 University International College, Macau University of Science and Technology, Macau 999078, China; 3230001579@student.must.edu.mo; 2 School of Foreign Languages, Zhejiang University of Finance and Economics Dongfang College, Haining 314408, China; 2120800419@zufedfc.edu.cn (X.X.); 20060029@zufedfc.edu.cn (Y.Z.)

**Keywords:** online TCFL teachers, teacher digital competence, teacher psychological resilience, teacher self-efficacy, structural equation modeling

## Abstract

In the context of the increasing digitization of education, online teachers’ competence and the affective factors of digital competence, psychological resilience, and self-efficacy have become prominent areas of academic inquiry. However, there is a dearth of extant research addressing the interactions between these variables in an online teaching Chinese as a foreign language (TCFL) environment. This study investigated the relationships among the digital competence, psychological resilience, and self-efficacy of online TCFL teachers via quantitative research methods. The questionnaires were administered to 610 online TCFL teachers, and the data were analyzed via structural equation modeling. The results indicate that digital competence and psychological resilience can significantly predict online teachers’ self-efficacy, and that the relationship between psychological resilience and self-efficacy can be significantly moderated by digital competence. This finding not only provides novel perspectives and insights for enhancing online teaching effectiveness, particularly in the domain of online foreign language instruction, but also complements and extends self-efficacy theory from the perspective of social cognitive theory.

## 1. Introduction

With the development of positive psychology, foreign-language teachers’ psychology has emerged as a significant area of international concern (M. Wang et al., 2022; Namaziandost et al., 2023; Liu et al., 2024). Positive psychological variables, such as teacher self-efficacy (TSEF) (Polin, 2023) and teacher psychological resilience (TPR) (Zhi & Derakhshan, 2024), have become central to recent research alongside the growing emphasis on teacher digital competence (TDC) in response to the rapid development of information and communication technology (ICT) (Rahimi, 2024). However, research on these three variables has focused primarily on offline and traditional classroom settings (Feng, 2024).

Twenty-first-century digital transformation has profoundly reshaped the educational landscape, leading to a significant increase in online teaching and learning (Kumar et al., 2017). Consequently, scholarly interest in self-efficacy, psychological resilience, and digital competence has gradually expanded from traditional face-to-face settings to online learning contexts. However, existing research has focused primarily on online learners rather than on teachers. Shen et al. (2013) identified five dimensions of self-efficacy in online learning and established that the number of online courses taken, gender, and academic status are significant predictors of self-efficacy in online learning. More recently, S. Wang et al. (2024a) explored the relationships among psychological resilience, online learning performance, academic burnout, and satisfaction, and revealed that psychological resilience was positively associated with online learning performance and satisfaction but negatively correlated with academic burnout. Feng (2024) reported that digital competence strongly predicts online learners’ perceived enjoyment and self-efficacy in foreign language learning. While these studies provide valuable insights into learners’ experiences, they fail to address how psychological resilience, self-efficacy, and digital competence interact in an online teaching context, particularly among foreign language instructors.

The drive toward digitalization and a shift toward online teaching has also highlighted the importance of the psychological dimension (Lan & Bin Saad, 2024) and digital competence (Moorhouse, 2023) of language teachers in online environments. Consequently, research on online teaching should expand (Dong et al., 2023), particularly considering the pivotal role of the teaching environment in the assessment of the TSEF (Tschannen-Moran & Hoy, 2001). However, research on TSEF in online classrooms remains limited compared with studies focused on traditional face-to-face settings. Corry and Stella (2018) argued that teaching efficacy in online environments should be treated as a distinct area of study given the significant differences between online and traditional classrooms. Furthermore, teachers’ psychological resilience is subject to changes in response to environmental changes. Consequently, it is necessary to reassess psychological resilience in online environments (Beltman et al., 2011) and effectively manage teachers’ emotions while maintaining the efficacy of online teaching (Peng & Hu, 2024). In parallel, TDC has attracted increasing research interest, particularly in relation to self-efficacy. Self-efficacy explains differences in teachers’ digital competence and ICT use, with strategies to evaluate information being key to developing digital competence (Hatlevik, 2017). Using the technology acceptance model, perceived usefulness, ease of use, attitudes, and self-efficacy significantly influence technology acceptance (Al-Hattami, 2023), and digital accounting tools can promote innovation, with technological self-efficacy enhancing its impact, whereas digital literacy does not significantly moderate this relationship (Al-Hattami, 2025). These studies underscore the importance of self-efficacy in shaping digital competence, technological acceptance, and innovation. However, they have overlooked the mediating role of digital competence.

Additionally, the interrelationship between TCFL teachers’ digital competence and affective factors remains an underexplored area of research. Existing studies indicate that teachers with greater digital competence often demonstrate greater confidence and beliefs in their anticipated performance (Sang et al., 2023). Moreover, teachers’ psychological traits and personal characteristics are linked to varying levels of self-efficacy (Xiyun et al., 2022). The TPR, conceptualized as a psychological state, may influence self-efficacy. However, the connection between the TPR and TDC is not well understood. While prior research has examined these factors individually, their interdependencies in the context of online TCFL teaching remain largely unexplored.

Although recent studies have begun to focus on TPR in online teaching and learning environments (Beltman et al., 2011; Peng & Hu, 2024), the need to remeasure psychological resilience in online settings has not been adequately addressed. In addition, online courses for second-language learners of Chinese have become a popular route to language acquisition (Guo et al., 2024), and research on online teachers, especially their digital competence, remains insufficient (Dong et al., 2023). This study is necessary because digital competence, self-efficacy, and psychological resilience are crucial factors influencing online foreign language teachers; however, the interrelationships among these variables have not been adequately explored.

This study aims to fill this gap by examining the interactions among TSEF, TPR, and TDC among online TCFL teachers. This study has theoretical and practical implications. On a theoretical level, this study expands and complements Bandura’s theory of self-efficacy and positive psychology; on a practical level, this study provides guidance for teacher training, educational policymaking, and the professional development of foreign language teachers. First, online foreign language teacher training can draw on the results of this study to develop effective training programs that enhance psychological resilience, self-efficacy, and digital competence. Second, educational policymakers can use the results of this study to promote the psychological development and digital competence of foreign language teachers and to provide support for their professional development. Finally, online foreign language teachers can use this study to gain a deeper understanding of psychological resilience, self-efficacy, and digital competence and how they affect teaching practices to guide their teaching behavior more effectively.

### 1.1. Teacher Self-Efficacy

**Figure 1:**
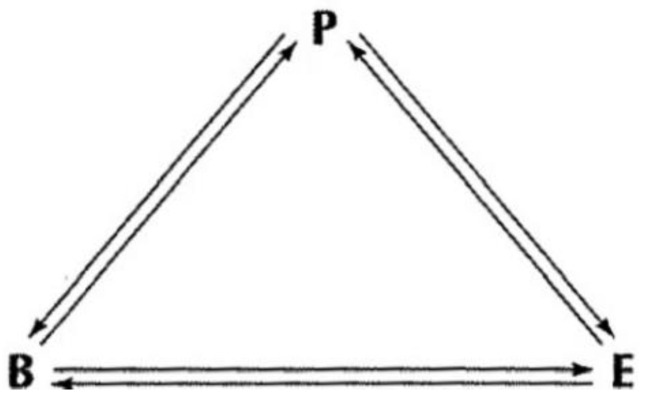
Bandura’s triad reciprocal determinism (Bandura, 1986).

Bandura’s (1986) social cognitive theory states that individuals’ behavior is shaped by triadic reciprocal determinism, whereby personal factors (e.g., cognition and emotion), behavior, and the environment interact with each other. Social cognitive theory asserts that people’s thoughts, beliefs, and feelings influence their behavior, which is related to personal factors and the environment interacting with each other, as illustrated in Figure 1. In the context of this theory, variables B, P, and E represent behavior, the subject, and the environment, respectively. The bidirectional arrows indicate a mutually deterministic relationship between these factors. In a classroom setting, these interactions are key to the study of complex relationships among teachers, instruction, and students.

The term “self-efficacy” is used to describe an individual’s perception or belief regarding their capacity to adopt adaptive behaviors in response to environmental change. It represents a comprehensive expression of an individual’s self-concept and self-evaluation, encompassing a range of psychological feelings such as self-confidence, trust, and self-esteem. The concept of self-efficacy not only produces psychological responses to behaviors that have already occurred in an individual, but also has a significant effect on subsequent behaviors. This demonstrates the ability to predict individual behavior (Bandura, 1977). Since the concept of self-efficacy was introduced into the field of education, the term ’teacher self-efficacy’ has been defined as a teacher’s confidence in their ability to successfully organize and implement a teaching task in a given context (Tschannen-Moran et al., 1998). Teacher self-efficacy has emerged as a pivotal indicator of educators’ perceptions of their capacity to fulfill their instructional responsibilities. It has been acknowledged as a fundamental element of teacher training programs (Perera et al., 2019). Bandura (1997) identified mastery and vicarious experiences gained by observing others, social persuasion, and emotional and physiological states as the four main sources of self-efficacy.

In the specific context of Chinese as a foreign language (CFL) online teaching, we defined TSEF as teachers’ self-beliefs and perceptions of their ability to impart Chinese language knowledge, use online technologies for effective online teaching and learning, and strategically engage and sustain student engagement in online teaching and learning activities. This definition encompasses not only teachers’ self-assessment of their professional competence, but also their confidence in using modern educational technologies, their self-assessment of teaching interactions, and their engagement in online teaching environments.

TSEF represents a pivotal aspect of students’ motivation and commitment to teaching; consequently, it has become a focal point of attention within the field of educational research (Polin, 2023). Self-efficacy is of pivotal importance in teachers’ personal goal setting, resilience in the face of challenges, and the implementation of teaching behaviors, including the utilization of digital teaching resources (Glackin & Hohenstein, 2018). In recent years, there has been a notable increase in research on online TSEF, particularly with the advent of online technology. For example, Liu et al. (2021) conducted an in-depth study on the self-efficacy of English as a foreign language (EFL) for teachers (N = 486) in Chinese online live classes via a mixed research method that combined questionnaires and interviews. This study employed exploratory factor analysis to identify two dimensions of online TSEF: instructional self-efficacy and technological self-efficacy. The findings revealed that the self-efficacy of these EFL teachers was generally at a medium to high level, with technological self-efficacy being particularly prominent. Another survey was conducted with TCFL preservice teachers (N = 331) from two key universities in China using a questionnaire. Based on the collected data, a structural equation model (SEM) was constructed by combining the variables of technology self-efficacy, intention to use technology, perceived usefulness, and attitudes toward and experiences with technology. The findings indicate that perceived usefulness, technology self-efficacy, and facilitation have direct and significant positive effects on Chinese language teachers’ attitudes toward the use of technology (Sun & Mei, 2022).

The extant literature has demonstrated that teachers’ self-efficacy is not only associated with their individual pedagogical practice and professional development, but also constitutes a pivotal element in the promotion of educational technology and digital teaching. Considering the digital transformation of educational environments and the advent of online teaching, a comprehensive investigation of online teachers’ self-efficacy and its influencing factors is imperative.

### 1.2. Teacher Digital Competence

The term “digital competence” is typically used to describe an individual’s capacity to utilize ICT, including the manipulation of digital tools or software (Antonietti et al., 2022). While there is no consensus on a single definition of digital competence for teachers, there is a general consensus that it encompasses not only the ability to utilize technological devices and digital resources within an educational setting, but also the attitudes, strategies, and awareness of teachers in their pedagogical practices. This competence enables teachers to integrate technological tools efficiently, thereby achieving the desired pedagogical outcomes (Cattaneo et al., 2021). Furthermore, digital competence has been shown to positively affect teaching and learning efficiency. Foreign language teachers can reduce their workload, stress, and frustration while enhancing their productivity and organizational and time management skills by easily and quickly accessing, managing, and sharing information and data (Zhao et al., 2021). In some instances, digital competence is synonymous with digital literacy (Falloon, 2020). In this study, TDC is defined as teachers’ capacity to utilize digital technologies and tools to teach and research Chinese in an online teaching and learning environment. This encompasses not only the operational level of technology, but also teachers’ comprehensive utilization of instructional design, content delivery, and student interaction, reflecting their professional literacy and innovation in the digital teaching and learning environment.

Digital competence has emerged as a key objective in global educational innovation in the digital age. It is regarded as a cross-cutting core competency closely intertwined with a range of 21st-century skills, including linguistic competence, mathematical literacy, learning competence, and cultural awareness (Ferrari, 2013). Digital competence is a crucial and indispensable skill for those engaged in teaching Chinese as a foreign language online on an international basis. Specific requirements and standards for TDC have been proposed in several countries. Among them, the European Digital Competence Framework for Teachers is one of the most representative and widely used. The framework consists of three modules: professional competence, pedagogical competence, and learner competence. These modules cover six areas: professional engagement, digital resources, teaching and learning, assessment, empowering learners, and facilitating their digital competence (Antonietti et al., 2022; Romero-Tena et al., 2021).

The importance of TDC in online teaching and learner experience has been well recognized in existing research (Chen et al., 2024). Hatlevik (2017) revealed the relationships between basic ICT self-efficacy and online collaboration self-efficacy, information evaluation strategies, and digital literacy through SEM, and between the research language, literature, and applied linguistics, and explored the relationships between digital literacy and technology acceptance among Chinese second language teachers with different professional backgrounds, including Chinese language and literature, Chinese second language education, English language and literature, and applied linguistics. This study emphasizes that educators and policymakers should pay attention to the development and improvement of digital literacy and provide more digital literacy training and support for teachers to promote their professional development and improve the quality of education. However, whether digital literacy plays the same role in online Chinese language teaching and learning requires further research.

### 1.3. Teacher Psychological Resilience

Psychological resilience can be defined as an individual’s capacity to adapt positively and continue professional development when faced with challenging circumstances (Stavraki & Karagianni, 2020). Research findings suggest that the TPR has a positive effect on an individual’s psychological state and predicts other aspects of the teaching field, including career success, professional enthusiasm, and retention (Liu & Chu, 2022; Tait, 2008).

Some studies have noted the effects of psychological resilience on teachers’ psychological variables. Xie (2021) examined the predictive roles of emotion regulation and psychological resilience in Chinese EFL teachers’ work engagement. This study revealed that both psychological resilience and emotion regulation were significant predictors of work engagement. Furthermore, research has concentrated on variables that affect teachers’ psychological resilience. Y. Wang et al. (2024b) reported that both Chinese and Iranian EFL teachers consider human-centered factors to be the main challenges in maintaining teachers’ psychological resilience. Other factors, including system-centered, context-centered, and process-centered factors, are perceived to have relatively minor impacts on teacher psychological resilience. Other studies have employed teacher self-efficacy and psychological resilience as primary independent variables and have proposed their influence on teacher burnout, psychological adjustment, and psychological well-being. S. Li (2023) evaluated the relationships between TSEF, TPR, emotion regulation, and teacher burnout in a study of 638 EFL teachers. The structural validity of the measurement instrument was confirmed via validated factor analysis, and the relationships among these variables were subsequently assessed via SEM. The results indicated a negative relationship between teacher self-efficacy and psychological resilience and burnout, and that emotion regulation indirectly influenced burnout through psychological resilience. Zhi and Derakhshan (2024) investigated the interactions among adaptive capacity, emotion regulation, and psychological well-being among Chinese EFL teachers. Regression analyses revealed that Chinese TSEF mediates the interaction between emotion regulation, psychological resilience, and psychological well-being. Additionally, the study revealed a strong correlation between emotion regulation and the TPR (correlation coefficient, r = 0.88; sample size, n = 430; significance level, *p* < 0.001).

Psychological resilience, as a significant psychological resource for educators to navigate professional challenges, has a profound effect on professional growth and teaching efficacy. By bolstering the TPR, work engagement can be effectively enhanced, burnout can be mitigated, and psychological well-being can be promoted. However, the important role of online language teachers’ psychological resilience in their digital competence and self-efficacy deserves further investigation.

### 1.4. Relationships of Psychological Resilience and Digital Competence with Self-Efficacy Among Teachers

Numerous studies have highlighted various strategies to increase teacher self-efficacy and underscored the need for a more comprehensive exploration of their digital competence (Hatlevik, 2017). Research has increasingly recognized the interrelationship between teachers’ self-efficacy and their digital competence, suggesting that a deeper understanding of this relationship could inform targeted interventions aimed at improving teaching outcomes in technology-rich educational environments.

On the one hand, existing research provides evidence that TSEF serves as a significant predictor of digital competence development. For example, self-efficacy in basic ICT skills has been found to significantly predict numerical ability (β = 0.46, *p* < 0.01), indicating that teachers who perceive themselves as competent in ICT are more likely to develop higher levels of digital competence (Hatlevik, 2017). Similarly, Z. Wang and Chu (2023) identified a positive correlation between self-efficacy and digital competence among teachers, suggesting that favorable external conditions can either directly enhance digital competency or indirectly do so by influencing teachers’ perceptions of their own efficacy. Dai (2023) demonstrated a moderate positive correlation between ICT self-efficacy and digital competence in a study involving EFL preservice teachers (β = 0.43, ρ < 0.001), reinforcing the notion that self-perceived ICT competence is a critical factor in the development of digital skills.

On the other hand, teachers with higher levels of digital competence are more likely to frequently integrate digital technology into their teaching practices, which in turn enhances their confidence in achieving desired educational outcomes (Eastin & LaRose, 2006; Sang et al., 2023). Hammond et al. (2011) investigated the motivations behind teachers’ use of ICT and reported that teachers with low self-efficacy were “one of the least frequent ICT users” (p. 196). This finding suggests that the infrequent use of ICT or digital technology is often associated with low self-efficacy. Similarly, Ben Amotz et al. (2022) highlighted the importance of enhancing the online presence of digital competence development for physical education teachers to improve their professional efficacy, specifically their confidence in enabling students to achieve desired learning outcomes. For preservice teachers, the perception of their own digital competence has been shown to significantly influence their self-efficacy in managing classroom discipline and in promoting students’ use of ICT to support learning. Elstad and Christophersen (2017) argue that these perceptions play a crucial role in shaping instructional self-efficacy, particularly in technology-enriched classroom settings. This perspective aligns with the broader view that digital competence not only enhances teaching practices, but also reinforces teachers’ beliefs in their ability to manage and facilitate learning effectively.

A review of the literature reveals a robust correlation between teachers’ digital competence and self-efficacy, suggesting a dynamic and mutually reinforcing relationship. However, a significant gap remains: most studies have focused on traditional classroom settings or subjects outside the context of online education. In particular, the question of whether higher levels of digital competence among teachers translate into greater self-efficacy in the specific context of online teaching of Chinese as a foreign language has not been sufficiently addressed. Addressing this gap is essential for developing effective professional development programs to support online TCFL teachers:

**H1:**  *Online TCFL teachers’ digital competence is positively associated with their self-efficacy.*

Psychological resilience is a collection of positive psychological characteristics, including job satisfaction, professional commitment, teaching efficacy, intrinsic motivation, personal well-being, and professional identity (Mansfield, 2020). Research has consistently demonstrated a direct positive correlation between psychological resilience and teachers’ well-being, suggesting that resilience is a critical factor in maintaining and enhancing teachers’ mental health and job performance (Brouskeli et al., 2018; Kibe & Boniwell, 2015; Zhi & Derakhshan, 2024). Notably, psychological resilience not only predicts teachers’ well-being, but also significantly predicts their psychological well-being. For example, Han (2022) conducted an online survey of 343 EFL teachers and reported that psychological resilience played a pivotal role in enhancing their psychological well-being, thereby highlighting the importance of resilience in coping with the demands of teaching. Q. Li et al. (2019) further suggested that TPR comprises self-efficacy, professional commitment, intrinsic motivation, and job fulfillment. Zhi and Derakhshan (2024) investigated the predictive role of psychological resilience and emotion regulation on EFL teachers’ psychological well-being. This was achieved through confirmatory factor analysis (CFA), correlation analysis, and multiple linear regression (MLR) analysis. The findings indicated that TSEF acted as a mediator in the relationship between psychological resilience and well-being (β = 0.47, *p* < 0.002) and between emotion regulation and psychological well-being (β = 0.65, *p* < 0.001).

Moreover, resilience has been shown to fully mediate the relationship between basic psychological needs and engagement, serving multiple functions, such as enhancing adaptability, promoting persistence, buffering stress, and fostering self-efficacy (Gao et al., 2025). Gao et al. (2025) argued that resilience instills a sense of determination, motivating individuals to persist through the challenges encountered during the learning process. This resilience-driven perseverance sustains continuous engagement and commitment to learning, enabling individuals to view obstacles as stepping stones rather than barriers. In digital teaching environments, resilience has the potential to bolster teachers’ persistence and adaptability significantly, thereby supporting ongoing professional development and effective instructional practices.

Although the interaction between TPR and self-efficacy has been substantiated to a certain extent in traditional offline classroom settings, particularly in EFL teaching contexts, the nature of this relationship in online teaching and learning environments remains underexplored. This gap is particularly evident in the field of international Chinese language teaching, where the unique challenges of online platforms may influence the dynamics of resilience and self-efficacy differently. Investigating this relationship is essential for understanding how to support online teachers effectively in maintaining their well-being and teaching effectiveness. On the basis of the gap in the literature and the theoretical framework suggesting a significant role of resilience in enhancing self-efficacy, this study proposes the following hypothesis:

**H2:**  *Online TCFL teachers’ psychological resilience is positively associated with their self-efficacy.*

Digital competencies, often referred to as digital skills, are defined as the ability to acquire, process, and apply knowledge effectively in practical and educational contexts. Teachers who possess these competencies are essential for communicating effectively, building meaningful connections, and facilitating cognitive and affective interactions in digital learning environments (Brauer, 2019). Research has shown that inadequate mastery of TDC may lead to negative emotional states, underscoring the importance of developing robust digital skills to mitigate stress and enhance teaching efficacy (Ben Amotz et al., 2022). In a recent study, Anthonysamy (2023) used a questionnaire survey and statistical analyses involving 563 Malaysian university students to examine the impact of metacognitive strategies on psychological resilience, digital literacy, and competence. The findings revealed that learners who employed metacognitive strategies presented greater psychological resilience and positive digital literacy and competence outcomes (β = 0.554). These results suggest that enhancing digital competence can contribute significantly to psychological resilience and academic performance, and position resilience as a core component of sustainable digital education practices. Feng (2024) reported that digital literacy, which is frequently equated with digital competence (Falloon, 2020), plays a pivotal role in second language acquisition. This finding was validated through a questionnaire survey and SEM, which verified the predictive role of students’ digital literacy on perceived enjoyment and self-efficacy in online learning.

Studies have shown that university-organized remote learning can significantly enhance student psychological resilience (Appolloni et al., 2021). However, when students have negative experiences with digital technologies, they often struggle to continue learning online (Eri et al., 2021). This challenge is largely attributed to the fact that most universities are unprepared for a complete transition to fully digital learning environments, leading to increased perceived difficulties among students (Houlden & Veletsianos, 2020). These findings imply that students with higher levels of psychological resilience are better equipped to navigate and overcome these challenges, allowing them to continue online learning more effectively. Consequently, by successfully managing the stress and difficulties associated with digital platforms, students can further develop their digital competence. This suggests a reciprocal relationship in which psychological resilience not only helps sustain engagement in online learning, but also facilitates the acquisition and enhancement of digital competence.

By extension, this relationship can also be expected to hold for teachers. Psychological resilience, as one of the essential competencies for teachers to adapt to and cope with the challenges of online teaching, plays a pivotal role in online foreign language teaching in Chinese. However, the relationship between digital competence and psychological resilience has been relatively underexplored in the context of online foreign language teaching. Most existing research has focused primarily on learners, leaving a substantial gap in the understanding of how these competencies interact within the teacher community. Teachers with greater psychological resilience are likely to manage digital challenges more effectively, thereby enhancing their digital competence in an online teaching environment. This theoretical perspective highlights the need to further investigate this relationship and provides strong support for the following hypotheses:

**H3:**  *Online TCFL teachers’ psychological resilience is positively associated with their digital competence.*

S. Li (2023) studied the effects of TSEF, TPR, and emotion regulation on teachers’ burnout and reported that TPR acted as a mediator, allowing emotion regulation to indirectly influence teachers’ burnout. However, Galindo-Domínguez et al. (2020) analyzed TPR as a dependent variable via statistical methods, such as correlation analysis, with a sample of 384 participants. Their findings indicated that while the TPR contributes to self-efficacy, it does so indirectly rather than exerting a direct influence. This implies that other factors may mediate the effect of the TPR on self-efficacy, highlighting the complexity of the relationships between these constructs.

Research has also demonstrated that digital competence serves as a mediating variable between strategies for evaluating information and ICT use as well as between teachers’ self-efficacy and ICT use (Hatlevik, 2017). Higher levels of digital competence enable teachers to use technology more critically and reasonably, which reinforces their confidence and self-efficacy (Krumsvik, 2014). This mediating role of digital competence suggests that enhancing teachers’ digital skills could indirectly increase their self-efficacy by improving their ability to effectively integrate technology into their teaching practices.

Despite the recognized importance of these psycho-emotional factors, the interaction between digital competence and psycho-emotional factors such as TPR and self-efficacy has largely been neglected in the literature. Most existing studies have focused on the dimensions, models, and conceptualizations related to digital terminology without examining the underlying psychological mechanisms (Feng, 2024). As a result, the relationships among TDC, TPR, and self-efficacy remain unclear, especially for online TCFL teachers. Addressing this gap is essential in developing comprehensive strategies to increase teachers’ effectiveness and well-being in digital learning environments. Accordingly, this study proposes the following hypotheses:

**H4:**  *Online TCFL teachers’ digital competence mediates the relationship between their psychological resilience and self-efficacy.*

**Figure 2:**
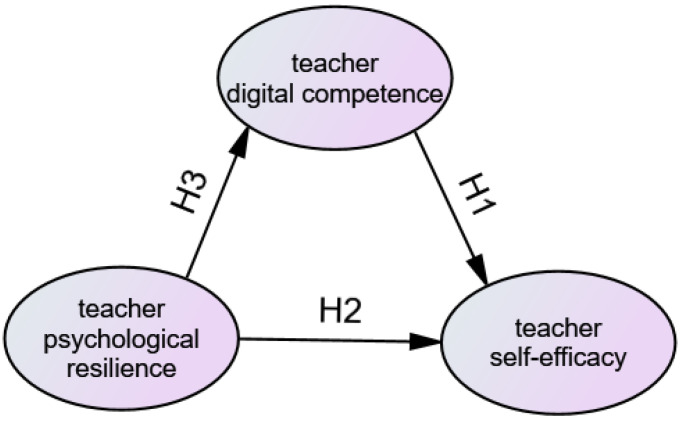
Research model hypothesis.

The principal objective of this study was to examine the interconnections between digital competence, psychological resilience, and self-efficacy among online TCFL teachers. In particular, it seeks to investigate the impact of TDC and TPR on TSEF with the aim of elucidating the distinctive challenges faced by online TCFL teachers and promoting their psychological development. This study contributes to psychology and competence research on foreign language teachers. The hypothesized model used in this study is shown in Figure 2.

## 2. Materials and Methods

### 2.1. Participants

In total, 610 online TCFL teachers participated in this study. To ensure data quality, responses with excessively short completion times (under 100 s) were excluded, as preliminary testing indicated that such durations likely reflect insufficient engagement with the survey content. Additionally, responses were removed if they exhibited inconsistency or no discernible variation in indicator answers, quantified as providing uniformly homogeneous responses (e.g., consistently selecting only two, three, four, or five across all indicators). After these exclusions, the final valid sample comprised 525 teachers.

All the participants were native Chinese speakers who taught K-12. Among the respondents, 214 (40.8%) were male, and 311 (59.2%) were female. The age distribution was as follows: 83 teachers (15.8%) were under 25 years, 193 (36.8%) were between 26 and 30 years, and 249 (47.4%) were over 31 years.

### 2.2. Instrument

#### 2.2.1. Teacher Self-Efficacy Scale

The Self-Efficacy Scale for Online International Chinese Teachers was adapted from Lin and Zheng’s (2015) Self-Efficacy Scale and consists of two sections: instructional self-efficacy and technology self-efficacy (Cronbach’s alpha >0.6). The scale includes 14 self-reported items designed to assess teachers’ beliefs regarding their ability to achieve the desired instructional outcomes. The participants rated their level of agreement on a 5-point Likert scale, where 1 represented strong disagreement and 5 represented strong agreement. The scale has been validated and shown to be reliable, with Cronbach’s alpha ranging from 0.772 to 0.873, as confirmed in previous studies, such as (Liu et al., 2021). These studies provide supporting evidence for the factor structure and psychometric properties of the scale, validating its validity in assessing the TSEF, which is translated in this paper, with the inclusion of item 7. I am able to improve my teaching effectiveness even though my cultural background is different from that of my students, as the foreign language teachers explored in this paper are all Chinese teachers, and the students are all overseas CFL learners.

#### 2.2.2. Teacher Psychological Resilience Scale

The online TPR scale was derived from the Connor–Davidson Resilience Scale Short Version (CD-RISC-10), which is a simplified adaptation of the original CD-RISC developed by Campbell-Sills and Stein (Campbell-Sills & Stein, 2007). This version was combined with the Chinese adaptation of the CD-RISC by Yu and Zhang (2007) to create the final scale. The final scale demonstrated strong reliability, with a Cronbach’s α of 0.85 and a determinacy value of 0.93 for the resilience factor. The scale comprises 10 items and is scored on a 5-point Likert scale ranging from 1 (indicating “not at all like this”) to 5 (indicating “almost always like this”), with higher scores indicating higher levels of psychological resilience. The scale has been demonstrated to possess favorable psychometric properties and has been validated to have high reliability and validity (Lauridsen et al., 2017). Furthermore, a psychological resilience score of less than 60 indicates poor resilience, a score between 60 and 69 indicates fair resilience, a score between 70 and 79 indicates good resilience, and a score of 80 or above indicates excellent resilience (Zhang et al., 2024).

#### 2.2.3. Digital Competence of Teachers Measurement

The digital competency scale was adapted from the version by Chen et al. (2024), which was based on a revised scale originally designed for college students by Hong and Kim (2018). Chen et al. (2024) modified this scale to assess digital competency in the context of online teaching for teachers. The scale was validated as an effective tool for online educators, demonstrating strong reliability (Cronbach’s α = 0.86–0.97), good model fit in confirmatory factor analysis, and robust validity, with factor loadings, composite reliability, and AVE values surpassing the recommended thresholds. The scale comprises 17 items organized into five dimensions: (1) digital tool use in teaching (DTUT), (2) digital application use in teaching (DAUT), (3) digital media safety awareness (DMSA), (4) digital resource search and integration skills (DRSIS), and (5) utilization of digital technology for interaction and collaboration (UDTIC). The paper was translated into Chinese and scored on a 5-point Likert scale, with 1 indicating strong disagreement and 5 indicating strong agreement.

### 2.3. Procedure and Data Collection

The data collection phase of this study was conducted between June and July 2024. A random sampling method, including simple random sampling and snowball sampling, was employed to recruit TCFL teachers engaged in online teaching. These techniques were used to ensure sample diversity. Recruitment information was posted on mainstream online platforms, including WeChat, Weibo, and REDnote. The questionnaire was distributed via these platforms, and teachers who were willing to participate were required to present proof of being online TCFL teachers, such as their employee ID cards. On average, the participants took approximately 5 min to complete the questionnaire. To express gratitude for their time and participation, the participants received an online cash transfer as a reward. These measures were implemented to enhance the authenticity and reliability of the collected data and thereby improve the validity and generalizability of the research findings.

The questionnaire was translated and jointly proofread by two experts proficient in both Chinese and English to ensure its accuracy and consistency. The participants contacted the researchers directly via the recruitment message, and upon understanding the purpose of the survey, they were invited to complete an online questionnaire linked to https://www.wjx.cn. (accessed on 8 July 2024). Prior to completing the questionnaire, the participants were required to read and agree to an informed consent form, thereby ensuring that all the data collected were based on the teachers’ fully informed and voluntary participation.

The participants were explicitly informed that they could withdraw from the study at any time during the data collection process if they felt uncomfortable and were assured that all the data would be kept strictly confidential and used for academic research purposes only.

We adhered to several ethical principles throughout the data collection process. These include the principles of full disclosure and voluntary participation, which are based on the morality and legitimacy of the study (Mackey & Gass, 2016).

### 2.4. Data Analysis

Prior to data analysis, questionnaires deemed invalid or containing logical contradictions were excluded to ensure data quality and reliability. The data were then processed via SPSS 26.0 for descriptive statistics and correlation analyses and AMOS 26.0 for partial least squares structural equation modeling (PLS-SEM). SPSS was chosen for basic statistical analyses, whereas AMOS was selected for managing complex path analyses in PLS-SEM, allowing for a more accurate assessment of the relationships among variables.

Before proceeding with SEM, the assumption of normality was evaluated on the basis of Byrne (2012), which considers data to be approximately normal if the absolute value of skewness is less than 2 and the absolute value of kurtosis is less than 5. The normality test is crucial for ensuring the scientific rigor and credibility of the conclusions.

Reliability and validity tests were performed to validate the measurement model. Validity was assessed through CFA via AMOS 26.0 to confirm the factor structure of the latent constructs, including digital competence, psychological resilience, and self-efficacy. CFA was chosen for its ability to test whether the observed data fit the hypothesized factor structure, thus ensuring the construct validity of the measurement model.

To assess the fit of the empirical data to the hypothetical model, the following goodness-of-fit metrics were used: χ^2^/df ≤ 5, GFI ≥ 0.90, CFI ≥ 0.90, NFI ≥ 0.90, TLI ≥ 0.90, RMSEA ≤ 0.08, and SRMR ≤ 0.10. These metrics were selected on the basis of the criteria outlined by Hair et al. (1987) and Liu et al. (2024), which provide scientific standards and references for evaluating model fit. Through a comprehensive examination of these indicators, this study evaluated the model fit and ensured the reliability and validity of the research results (Hair et al., 1987; Liu et al., 2024).

## 3. Results

### 3.1. Descriptive and Correlation Analyses

Descriptive and correlation analyses were conducted for each variable involved in the study, and the results are presented in Table 1. The psychological resilience score for online TCFL teachers was 3.989 out of 5, whereas the TDC score was 4.067 out of 5. Furthermore, the participants had a self-efficacy score of 4.085 out of 5, which is indicative of a high level of this dimension. The means and standard deviations of the descriptive statistics provided insight into the concentration trends and dispersion of the scale data, whereas the skewness and kurtosis indicators were used to assess the normality of the data distribution.

The data distribution can be considered normal when the absolute value of skewness is less than two and the absolute value of kurtosis is less than five (Byrne, 2012). The absolute values of the skewness of the scale data presented in Table 1 did not exceed two, and the absolute values of kurtosis did not exceed five. This indicates that the collected data satisfied the assumption of a normal distribution. The results of this normality test confirmed the applicability of the subsequent analytical methods and the reliability of the findings.

### 3.2. Evaluation of the Measurement Model

A scale or questionnaire is considered to have good reliability if the Cronbach’s alpha coefficient is above 0.80 and acceptable if it is between 0.70 and 0.80. For the subscales, a coefficient above 0.70 is preferable, and 0.60 to 0.70 is acceptable. In this study, the Cronbach’s alpha coefficients for all the subscales were above 0.80, indicating that the questionnaire had good reliability (see Table 2).

As shown in Table 3, the fit indices for TPR, TDC, and TSEF were satisfactory: the χ^2^/df values were less than 3; the RMR and RMSEA values were less than 0.08; and the CFI, TLI, IFI, and GFI indices were all greater than 0.90. These results suggest that the construct validity of the three scales is good, meaning that they accurately measure the intended constructs.

Table 4 shows that the factor loadings for all items in the PR scale are above 0.50, indicating that these items effectively represent their respective latent variables. Additionally, the average variance extracted (AVE) values exceed 0.50, and the composite reliability (CR) values are above 0.70, confirming that the convergent validity of the TPR scale is satisfactory.

The TDC scale also demonstrated good convergent validity. The factor loadings for all dimensions (DTUT, DAUT, DMSA, DRSIS, and UDTIC) were above 0.50, the AVE values exceeded 0.50, and the CR values were above 0.70. This suggests that the items effectively represented their respective latent variables.

Similarly, the TSEF scale has yielded satisfactory results. The factor loadings for the ISE and TSE were above 0.50, the AVE values exceeded 0.50, and the CR values were above 0.70, indicating good convergent validity.

In summary, the scales showed high reliability, construct validity, and convergent validity, indicating that the measurement model is robust and reliable for further analysis.

### 3.3. Results of the Structural Equation Modeling

**Figure 3:**
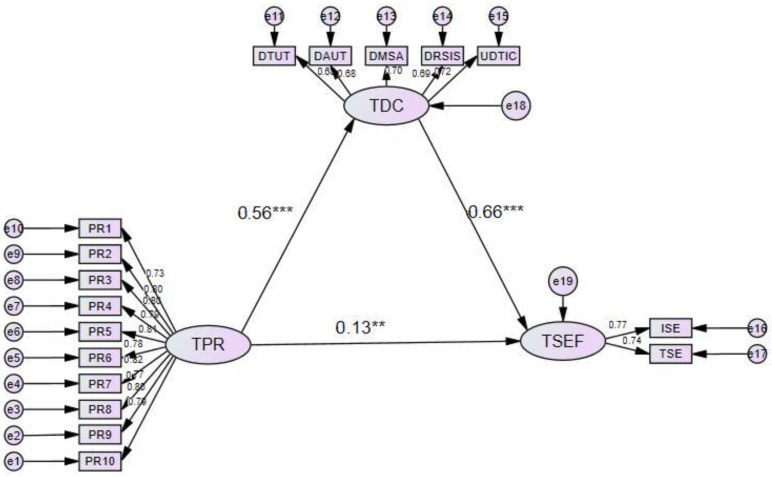
Model of the mediating role of the digital competence of teachers in the relationship between teacher self-efficacy and teacher psychological resilience. Note: TPR: teacher psychological resilience; TDC: teacher digital competence; TSEF: teacher self-efficacy. ** *p* < 0.01, *** *p* < 0.001.

In this study, SEM was employed to investigate the direct and potential indirect pathways through which TSEF was influenced by TPR and TDC. Figure 3 shows the SEM path diagrams with standardized coefficients. The X^2^/df ratio was 1.452, which was below the standardized threshold of 3. Similarly, the root mean square of residuals (RMR) was 0.013, and the root mean square error approximation (RMSEA) was 0.029, both of which were below the recommended value of 0.08. Furthermore, the comparative fit index (CFI) was 0.990, the Tucker–Lewis index (TLI) was 0.988, the incremental fit index (IFI) was 0.990, and the goodness-of-fit index (GFI) was 0.965, all of which exceeded the goodness-of-fit criterion of 0.9. The results demonstrated that the constructed model exhibited an excellent fit with the data, as shown in Table 5.

In terms of the relationships between the variables (Table 6), TPR was significantly and positively associated with TDC (*p* < 0.05), with a standardized path coefficient of 0.564. This result suggests that higher levels of TPR are related to higher levels of TDC, thereby validating Hypothesis 3. Additionally, TDC was significantly and positively associated with self-efficacy (*p* < 0.05), with a standardized path coefficient of 0.661, indicating that greater TDC was linked to higher self-efficacy, thus supporting H1. Furthermore, TPR was significantly and positively related to self-efficacy (*p* < 0.05), with a standardized path coefficient of 0.129, suggesting that higher levels of PR correspond to greater self-efficacy, confirming H2.

In terms of the mediating influence (Table 7), this study conducted a mediation effect test using 5000 bootstrap iterations. The results show that the confidence interval for the indirect effect does not contain 0 (0.299, 0.464), with a *p* value less than 0.05, suggesting that TDC plays a mediating role in the relationship between TPR and TSEF, thereby validating H4. Furthermore, the percentage of mediation was approximately 74.45% (0.373/0.501 × 100), indicating a substantial indirect effect. The direct effect remained significant (*p* = 0.038), implying that the mediation was partial rather than full. According to Baron and Kenny’s (1986) classic approach, a significant direct effect does not preclude the existence of mediation, suggesting that the mediating pathway through TDC plays an important, but not exclusive, role in explaining the relationship between PR and self-efficacy.

## 4. Discussion

### 4.1. The Relationship Between Digital Competence and Self-Efficacy

The results of SEM analyses indicated that TDC directly and positively predicted self-efficacy. This finding is consistent with those of previous studies (Eastin & LaRose, 2006; Hammond et al., 2011; Sang et al., 2023). The effective use of technology means that teachers are able to access digital information efficiently and thus achieve a greater sense of self-efficacy (Fanni et al., 2013). Fanni et al. (2013) and Hatlevik (2017) also demonstrated a positive correlation between ICT and teachers’ self-efficacy, particularly in terms of digital technological competence. This suggests that TDC, through the effective use of computers and communication technologies to process, transmit, and store information, significantly impacts self-efficacy in online teaching.

In an online teaching model, all instructional activities are conducted online, with students interacting remotely with teachers and peers (Schmid et al., 2023). For a more interactive online learning environment, it is essential for TCFL teachers to develop digital competencies that enhance student engagement and support effective teaching. Key skills involve designing interactive learning experiences that capture students’ attention, smoothly managing online discussions, and addressing challenges related to digital technologies during lessons. In addition, teachers need the ability to use digital tools to provide timely feedback and support students’ learning beyond the classroom through continuous guidance and resources. Proficiency in fostering an inclusive and collaborative online environment is crucial. These competencies have been empirically proven to enhance teaching effectiveness in terms of both achievement and attitudes (Means et al., 2009; Schmid et al., 2023).

The positive relationship between TDC and self-efficacy can be attributed to the increased sense of control and competence that teachers experience when effectively using digital tools. Mastery experiences gained through the successful management of digital tasks provide teachers with a series of positive achievements, thereby reinforcing their self-efficacy. Additionally, a high level of digital competence reduces technostress, which enhances teachers’ psychological resilience and willingness to adopt innovative teaching methods. Social recognition and positive peer feedback further strengthened teachers’ confidence in their abilities. Moreover, emotional stability resulting from proficiency in handling digital technologies minimizes anxiety and contributes to a stronger sense of self-efficacy.

Schmid et al. (2023) reported that non-STEM disciplines tend to have lower effect sizes in terms of self-efficacy outcomes than STEM fields do. This could be partly due to anxiety or lack of experience with learning-oriented technology among non-STEM teachers, particularly language arts teachers. This suggests that language teachers’ understanding of and proficiency in learning-oriented technologies (i.e., digital competence) directly related to their self-efficacy. Addressing this gap through targeted professional development that enhances digital competence could be crucial for improving self-efficacy among non-STEM teachers.

Self-confidence and self-efficacy are related to an individual’s beliefs about his or her ability to perform certain tasks effectively (Bandura, 1977). In accordance with Bandura’s theory, self-efficacy comprises four principal sources of information. The four main sources of information that contribute to the formation of self-efficacy are mastery experiences, vicarious experiences gained by observing others, social persuasion, and emotional and physiological states. Digital competence may be conceptualized as a form of mastery experience given its direct correlation with teachers’ capacity to utilize digital tools effectively in the classroom. When teachers are able to teach effectively via digital tools, this success increases their self-efficacy. Furthermore, as triadic reciprocal determinism emphasizes the interaction between cognitive and individual factors, environmental factors, and the behavior itself, it is possible to explain, from this perspective, that teachers who are digitally competent and believe in their own ability to perform online teaching tasks in a particular context, such as online teaching, have stronger self-efficacy beliefs.

### 4.2. The Relationship Between Psychological Resilience and Self-Efficacy

Psychological resilience significantly related to TSEF, and this study revealed a positive relationship between TPR and TSEF. This suggests that teachers who are more resilient and can effectively adapt to and recover from challenges have a more positive self-assessment of their abilities, particularly in the demanding context of online teaching. This aligns with the findings of Zhi and Derakhshan (2024), who similarly argued that TPR directly enhances TSEF in online TCFL teachers.

In this context, TPR plays a crucial role in providing teachers with positive alternative experiences, especially during times of adversity. Resilience helps teachers endure and learn from challenging situations, reinforcing their belief in their capacity to overcome future difficulties. Teachers with high resilience tend to face stressors, such as online teaching challenges, with a more adaptive mindset, allowing them to recover quickly and view setbacks as opportunities for growth rather than failure (Mansfield et al., 2016).

Moreover, the ability to recover from failure and learn from it is a key characteristic of high TPR and contributes to an increased sense of self-efficacy. Teachers with high TPR are more likely to engage in problem-solving behaviors when faced with challenges, maintain an optimistic outlook, and take proactive steps to improve their teaching (Liu & Chu, 2022). This resilience, in turn, fosters a feedback loop where successes and positive adaptations further bolster self-confidence and self-efficacy.

Daniilidou et al. (2020) support this notion, indicating that teachers with high resilience are not only more optimistic, but also experience less stress and greater job satisfaction. These emotional and psychological benefits contribute to the enhancement of self-efficacy. In sum, TPR equips teachers with the psychological resources to navigate the complexities of their work, particularly in online environments, thereby fostering a stronger belief in their capabilities and enhancing their overall effectiveness as educators.

Moreover, resilient teachers are often more effective in managing the environments of their schools or institutions, which can help alleviate feelings of anxiety and stress. Consequently, they experience lower levels of tension and are more likely to experience a stronger sense of unity within their professional community. This heightened sense of cohesion fosters a greater sense of belonging, which in turn enhances their self-confidence and belief in their ability to meet professional standards (Beltman et al., 2011). TPR not only helps teachers cope with challenges in the short term, but also plays a crucial role in sustaining long-term mental health and job satisfaction. This ability to manage stress and maintain well-being is an essential contributor to their continued sense of self-efficacy, creating a positive feedback loop where resilience supports self-efficacy (S. Li, 2023).

### 4.3. The Relationship Between Psychological Resilience and Digital Competence

TPR is positively associated with TDC, suggesting that the more positively adaptive online TCFL teachers are when faced with challenging situations, the more pronounced their ability to use digital technology is. Psychological resilience plays a crucial role in enhancing teachers’ digital competence, particularly in online teaching. Teachers with higher levels of resilience are better equipped to face challenges that arise with the rapid pace of technological change in education. These teachers demonstrate greater adaptability and persistence, allowing them to effectively overcome obstacles related to digital teaching. Rather than being discouraged by issues such as technical difficulties or complex online teaching environments, they approach such challenges as opportunities for growth. This aligns with the findings of Anthonysamy (2023), who reported that resilience positively affects learners’ digital competence and extends this relationship to the population of online TCFL teachers. The main reason for this phenomenon is that teachers with higher levels of TPR are more likely to demonstrate stronger self-efficacy in coping with work pressures, such as high workloads, difficult students, and challenging classroom environments (S. Li, 2023). In other words, teachers with higher TPR levels are more likely to fully master and effectively use their TDC in challenging online teaching environments.

Moreover, psychological resilience strengthens teachers’ emotional and cognitive capacity. Resilient teachers are better at managing stress, maintaining positive attitudes, and dealing with the emotional challenges that often accompany digital teaching. This emotional regulation helps them navigate the frustration of technical issues and online teaching complexities. They are also more open to feedback and actively seek ways to improve, which drives them to update their digital knowledge and skills to meet the evolving educational needs (Levano-Francia et al., 2019). This openness to learning, coupled with a willingness to experiment with new technologies, further enhances their digital competence. Resilient teachers are more likely to continue to explore and implement innovative teaching strategies, contributing to their long-term success in using digital tools.

Thus, TPR is not only related to TSEF but may also have a direct effect on TDC. Teachers with higher levels of mental toughness can quickly adapt their coping strategies and find and implement solutions when faced with technological difficulties and challenges in digital teaching and learning, thereby enhancing TDC. Additionally, they are more inclined to try new technologies and teaching methods, which helps them achieve continuous progress and innovation in the field of digital teaching. Therefore, strengthening the TPR is important for enhancing students’ digital teaching skills.

### 4.4. The Mediating Effect of Digital Competence on the Relationship Between Psychological Resilience and Self-Efficacy

The SEM results revealed the mediating role of TDC in the relationship between TPR and self-efficacy. Although previous studies have focused mostly on one or two of these three variables, such as the relationships between TDC and self-efficacy (Eastin & LaRose, 2006; Hammond et al., 2011; Hatlevik, 2017; Sang et al., 2023) and between TPR and self-efficacy (Q. Li et al., 2019; Zhi & Derakhshan, 2024), this study offers a more comprehensive understanding of how these three factors interact with online TCFL teachers.

Individuals with high levels of digital competence tend to be more familiar with the use and specification of new technologies, and their adaptation to new digital technologies significantly influences their cognitive load during use (Sang et al., 2023). This technological adaptability enables teachers to manage online teaching demands more effectively, thereby reinforcing their self-efficacy. This adaptability not only supports self-efficacy by enhancing teachers’ perceived competence, but also creates a foundation for continuous improvement. Moreover, high TPR equips teachers with the emotional and cognitive resilience needed to handle challenges, such as technical difficulties or instructional setbacks. This resilience helps teachers persist in developing their digital skills, thereby indirectly reinforcing their self-efficacy.

The relationships among TDC, TPR, and TSEF appear to be more complex than those in a simple cause-and-effect model. As discussed in Section 1.4, while previous studies have often explored how self-efficacy influences digital competence (Dai, 2023; Hatlevik, 2017; Z. Wang & Chu, 2023), the findings of this study suggest that the correlation between these factors is far from unidirectional. It is plausible that these variables influence each other in an intricate and reciprocal manner. For example, teachers with higher self-efficacy might be more inclined to use new digital tools, thereby enhancing their TDC, whereas improved digital competence could increase their self-efficacy. According to the triadic reciprocal determinism model (Bandura, 1977, 1997), external environmental factors, cognitive and personal factors, and behavioral events interact continuously and reciprocally. In the context of online teaching, TDC represents a critical competency that helps teachers effectively manage digital tools, reinforcing their self-efficacy. Simultaneously, the TPR enables teachers to cope with the complexities of online instruction, including technical challenges and intercultural communication. This reciprocal interaction between TDC, TPR, and self-efficacy suggests a more nuanced and interdependent dynamic that fosters both competence and resilience and promotes continuous professional growth.

The study’s findings highlight the significance of interactions among these factors in advancing online language teaching. TDC serves both as a product of environmental and behavioral engagement, and is a key factor that enhances teachers’ confidence in using digital technologies effectively. This increased confidence contributes to their instructional and technological self-efficacy, enabling them to manage digital tools more competently in their instructional practices. Moreover, the TPR enhances teachers’ ability to manage stress and recover from setbacks. This dynamic aligns with Bandura’s emphasis on the influence of social networks and external contexts on cultivating specific skills and values, especially in environments that demand both technological adaptation and intercultural sensitivity.

By integrating these elements and Bandura’s theory, this study highlights how TPR, TDC, and TSEF collectively shape teachers’ abilities to thrive in online teaching contexts. The findings of this study suggest that initiatives aimed at enhancing teachers’ digital competence should also address their psychological resilience, as the interplay between these factors is essential for sustained professional growth and effective teaching performance in the digital age.

## 5. Conclusions

This study employed SEM to provide an in-depth analysis of complex relationships among online TCFL teachers’ psychological resilience, self-efficacy and digital competence. This finding offers a novel perspective on the field of online foreign language teaching and the study of self-efficacy theory, while also providing significant guidance at both the theoretical and practical levels. This study not only extends the existing models of teacher self-efficacy, but also emphasizes the crucial role of enhancing teachers’ digital competence and information literacy in improving self-efficacy. Furthermore, it expands research on the psychological factors affecting foreign language teachers in the Chinese context, addressing the gap in research on how psychological resilience and digital competence, as independent variables, affect online TCFL teachers’ self-efficacy.

This study provides insights at both the theoretical and practical levels. At the theoretical level, this study enriches and develops Bandura’s theory of self-efficacy and proposes a triadic interaction theory in which personal factors, behavior, and the environment interact with each other, shedding deeper light on human behavior patterns. In the context of online TCFL teaching, TPR, TDC, and TSEF are core personal factors, and their interactions have a significant effect on teachers’ beliefs about teaching and the smooth running of online teaching activities. At a practical level, relevant platforms and institutions should first develop and implement training programs for online TCFL teachers, with a special emphasis on TDC. As TDC can predict self-efficacy, individual teachers should actively improve their TDC to adapt to the high demand for TDC in online teaching (Sang et al., 2023). Second, platforms and organizations should pay attention to teachers’ emotional needs and provide timely psychological counseling to enhance their ability to cope with the challenges of online teaching, thus improving their self-efficacy.

However, this study has some limitations. First, although this study constructed a path model illustrating the relationships between TDC, TPR, and TSEF, the specific and complex causal relationships among these variables remain uncertain and require further experimental validation. Future research could incorporate experimental designs to examine causality more rigorously and to clarify the direction and strength of these interactions. Second, regarding the factors influencing online international Chinese teachers’ self-efficacy, qualitative research methods such as in-depth interviews could be employed to explore in greater detail how teachers’ psychological resilience and digital competence affect self-efficacy. Additionally, future studies could introduce additional relevant variables, such as emotion regulation, emotional intelligence, and professional identity, to broaden our understanding of the mechanisms that influence teachers’ self-efficacy.

## Figures and Tables

**Table 1:** Descriptive statistics and correlations among latent variables.

	TPR	DTUT	DAUT	DMSA	DRSIS	UDTIC	TDC	ISE	TSE	TSEF
TPR	1									
DTUT	0.373 **	1								
DAUT	0.392 **	0.453 **	1							
DMSA	0.376 **	0.528 **	0.444 **	1						
DRSIS	0.376 **	0.460 **	0.458 **	0.471 **	1					
UDTIC	0.386 **	0.473 **	0.498 **	0.497 **	0.535 **	1				
TDC	0.496 **	0.784 **	0.730 **	0.758 **	0.751 **	0.798 **	1			
ISE	0.365 **	0.384 **	0.410 **	0.395 **	0.385 **	0.412 **	0.517 **	1		
TSE	0.375 **	0.361 **	0.405 **	0.367 **	0.377 **	0.361 **	0.486 **	0.571 **	1	
TSEF	0.418 **	0.420 **	0.460 **	0.429 **	0.430 **	0.435 **	0.566 **	0.881 **	0.892 **	1
Men	3.989	4.001	4.119	4.023	4.060	4.130	4.067	4.072	4.097	4.085
Std. Devition	0.679	0.655	0.661	0.669	0.666	0.629	0.502	0.603	0.631	0.547
Skewness	−0.489	−0.281	−0.521	−0.262	−0.348	−0.401	−0.290	−0.474	−0.564	−0.467
Kurtosis	−0.128	−0.542	−0.284	−0.650	−0.500	−0.563	−0.345	−0.336	−0.197	−0.247

Note: N = 525. TPR: teacher psychological resilience, DTUT: digital tool use in teaching, DAUT: digital application use in teaching, DMSA: digital media safety awareness, DRSID: digital resource search and integration skills, UDTIC: utilization of digital technology for interaction and collaboration, TDC: teacher digital competence, ISE: instructional self-efficacy, TSE: technological self-efficacy, TSEF: teacher self-efficacy. ** *p* < 0.01.

**Table 2:** Reliability test results.

Variable	α	Items
TPR	0.924	10
DTUT	0.843	4
DAUT	0.829	3
DMSA	0.793	3
DRSIS	0.817	3
UDTIC	0.850	4
TDC	0.912	17
ISE	0.903	7
TSE	0.912	7
TSEF	0.925	14

Note: TPR: teacher psychological resilience; DTUT: digital tool use in teaching; DAUT: digital application use in teaching; DMSA: digital media safety awareness; DRSID: digital resource search and integration skills; UDTIC: utilization of digital technology for interaction and collaboration; TDC: teacher digital competence; ISE: instructional self-efficacy; TSE: technological self-efficacy; TSEF: teacher self-efficacy.

**Table 3:** Construct validity.

Variable	Fitting Index	X^2^/df	RMR	RMSEA	CFI	TLI	IFI	GFI
TPR	Fitting values	2.263	0.014	0.049	0.988	0.985	0.988	0.972
TDC	Fitting values	1.420	0.016	0.028	0.989	0.986	0.989	0.966
TSEF	Fitting values	1.357	0.015	0.026	0.994	0.992	0.994	0.974

Note: TPR: teacher psychological resilience; TDC: teacher digital competence; TSEF: teacher self-efficacy.

**Table 4:** Convergent Validity.

Variable	Item	Factor Loading	S.E.	C.R.	*p*	AVE	CR
TPR	PR1	0.735				0.624	0.943
	PR2	0.793	0.069	18.434	***		
	PR3	0.801	0.061	18.622	***		
	PR4	0.793	0.061	18.435	***		
	PR5	0.809	0.062	18.830	***		
	PR6	0.776	0.059	17.994	***		
	PR7	0.825	0.061	19.229	***		
	PR8	0.772	0.060	17.898	***		
	PR9	0.798	0.059	18.559	***		
	PR10	0.793	0.058	18.413	***		
DTUT	DC1	0.759				0.579	0.846
	DC2	0.704	0.051	15.506	***		
	DC3	0.785	0.052	17.306	***		
	DC4	0.792	0.051	17.444	***		
	DC5	0.797					
DAUT	DC6	0.798	0.058	17.657	***	0.619	0.83
	DC7	0.765	0.053	17.072	***		
	DC8	0.742					
DMSA	DC9	0.769	0.092	15.626	***	0.575	0.802
	DC10	0.763	0.078	15.537	***		
	DC11	0.809					
DRSIS	DC12	0.753	0.055	16.787	***	0.598	0.817
	DC13	0.757	0.054	16.859	***		
	DC14	0.738					
UDTIC	DC15	0.799	0.064	17.214	***	0.587	0.85
	DC16	0.779	0.061	16.809	***		
	DC17	0.747	0.062	16.15	***		
SE	ISE1	0.740				0.574	0.904
	ISE2	0.746	0.066	16.887	***		
	ISE3	0.754	0.059	17.074	***		
	ISE4	0.771	0.059	17.497	***		
	ISE5	0.755	0.060	17.098	***		
	ISE6	0.767	0.058	17.407	***		
	ISE7	0.768	0.059	17.417	***		
TSE	TSE1	0.791				0.597	0.912
	TSE2	0.796	0.053	19.864	***		
	TSE3	0.811	0.054	20.332	***		
	TSE4	0.742	0.050	18.197	***		
	TSE5	0.757	0.053	18.628	***		
	TSE6	0.732	0.048	17.886	***		
	TSE7	0.777	0.049	19.256	***		

Note: TPR: teacher psychological resilience; DTUT: digital tool use in teaching; DAUT: digital application use in teaching; DMSA: digital media safety awareness; DRSID: digital resource search and integration skills; UDTIC: utilization of digital technology for interaction and collaboration; TDC: teacher digital competence; ISE: instructional self-efficacy; TSE: technological self-efficacy. *** *p* < 0.001.

**Table 5:** Test results of the degree of model fit.

Fitting Index	X^2^/df	RMR	RMSEA	CFI	TLI	IFI	GFI
Fitting values	1.452	0.013	0.029	0.990	0.988	0.990	0.965

**Table 6:** Different paths of the variables in the SEM.

Path			β	S.E.	C.R.	*p*
TPR	→	TDC	0.564	0.038	10.458	***
TDC	→	TSEF	0.661	0.074	9.285	***
TPR	→	TSEF	0.129	0.041	2.302	0.021 *

Note: TPR: teacher psychological resilience; TDC: teacher digital competence; TSEF: teacher self-efficacy. →: path direction, * *p* < 0.05, *** *p* < 0.001.

**Table 7:** Results of the mediation effect test.

Effect	Effect Size	95%CI		*p*
		Lower	Upper	
Direct effect	0.129	0.007	0.250	0.038 *
Indirect effect	0.373	0.299	0.464	0.000 ***
Total Effect	0.501	0.405	0.594	0.000 ***

Note: * *p* < 0.05, *** *p* < 0.001.

## Data Availability

The data presented in this study are available on request from the corresponding author. The data are not publicly available due to privacy.

## References

[B1-behavsci-15-00366] Al-Hattami H. M. (2023). Understanding perceptions of academics toward technology acceptance in accounting education. Heliyon.

[B2-behavsci-15-00366] Al-Hattami H. M. (2025). Understanding how digital accounting education fosters innovation: The moderating roles of technological self-efficacy and digital literacy. The International Journal of Management Education.

[B3-behavsci-15-00366] Anthonysamy L. (2023). Being learners with mental resilience as outcomes of metacognitive strategies in an academic context. Cogent Education.

[B4-behavsci-15-00366] Antonietti C., Cattaneo A., Amenduni F. (2022). Can teachers’ digital competence influence technology acceptance in vocational education?. Computers In Human Behavior.

[B5-behavsci-15-00366] Appolloni A., Colasanti N., Fantauzzi C., Fiorani G., Frondizi R. (2021). Distance learning as a resilience strategy during covid-19: An analysis of the italian context. Sustainability.

[B6-behavsci-15-00366] Bandura A. (1977). Social learning theory.

[B7-behavsci-15-00366] Bandura A. (1986). Social foundations of thought and action: A social cognitive theory.

[B8-behavsci-15-00366] Bandura A. (1997). Self-efficacy: The exercise of control.

[B9-behavsci-15-00366] Baron R. M., Kenny D. A. (1986). The moderator–mediator variable distinction in social psychological research: Conceptual, strategic, and statistical considerations. Journal of Personality and Social Psychology.

[B10-behavsci-15-00366] Beltman S., Mansfield C., Price A. (2011). Thriving not just surviving: A review of research on teacher resilience. Educational Research Review.

[B11-behavsci-15-00366] Ben Amotz R., Green G., Joseph G., Levi S., Manor N., Ng K., Barak S., Hutzler Y., Tesler R. (2022). Remote teaching, self-resilience, stress, professional efficacy, and subjective health among israeli PE teachers during the COVID-19 pandemic. Education Sciences.

[B12-behavsci-15-00366] Brauer S. (2019). Digital open badge-driven learning–competence-based professional development for vocational teachers. Doctoral thesis.

[B13-behavsci-15-00366] Brouskeli V., Kaltsi V., Loumakou M. (2018). Resilience and occupational well-being of secondary education teachers in Greece. Issues In Educational Research.

[B14-behavsci-15-00366] Byrne B. M. (2012). Structural equation modeling with Mplus: Basic concepts, applications, and programming.

[B15-behavsci-15-00366] Campbell-Sills L., Stein M. B. (2007). Psychometric analysis and refinement of the Connor-Davidson Resilience Scale (CD-RISC): Validation of a 10-item measure of resilience. Journal of Traumatic Stress.

[B16-behavsci-15-00366] Cattaneo A. A. P., Gurtner J.-L., Felder J. (2021). Digital tools as boundary objects to support connectivity in dual vocational education: Towards a definition of design principles. Developing connectivity between education and work.

[B17-behavsci-15-00366] Chen A., Li W., Fu W. (2024). Unleashing digital superheroes: Unravelling the empathy factor in digital competence and online teacher autonomy support. British Journal of Educational Technology.

[B18-behavsci-15-00366] Corry M., Stella J. (2018). Teacher self-efficacy in online education: A review of the literature. Research in Learning Technology.

[B19-behavsci-15-00366] Dai W. (2023). An empirical study on english preservice teachers’ digital competence regarding ICT self-efficacy, collegial collaboration and infrastructural support. Heliyon.

[B20-behavsci-15-00366] Daniilidou A., Platsidou M., Gonida S.-E. (2020). Primary school teachers’ resilience: Association with teacher self-efficacy, burnout and stress. Electronic Journal of Research in Educational Psychology.

[B21-behavsci-15-00366] Dong J., Zhang Y., Ma K. (2023). Online teaching self-efficacy of Chinese university teachers amidst Covid-19: Its changes and the moderation of adaptability and administration quality. Social Influence.

[B22-behavsci-15-00366] Eastin M. S., LaRose R. (2006). Internet self-efficacy and the psychology of the digital divide. Journal of Computer-Mediated Communication.

[B23-behavsci-15-00366] Elstad E., Christophersen K.-A. (2017). Perceptions of digital competency among student teachers: Contributing to the development of student teachers’ instructional self-efficacy in technology-rich classrooms. Education Sciences.

[B24-behavsci-15-00366] Eri R., Gudimetla P., Star S., Rowlands J., Girgla A., To L., Li F., Sochea N., Bindal U. (2021). Digital resilience in higher education in response to COVID-19 pandemic: Student perceptions from Asia and Australia. Journal of University Teaching and Learning Practice.

[B25-behavsci-15-00366] Falloon G. (2020). From digital literacy to digital competence: The teacher digital competency (TDC) framework. Educational Technology Research and Development.

[B26-behavsci-15-00366] Fanni F., Rega I., Cantoni L. (2013). Using self-efficacy to measure primary school teachers’ perception of ICT: Results from two studies. International Journal of Educational Development Using ICT.

[B27-behavsci-15-00366] Feng L. (2024). Modeling the contribution of EFL students’ digital literacy to their foreign language enjoyment and self-efficacy in online education. The Asia-Pacific Education Researcher.

[B28-behavsci-15-00366] Ferrari A., Yves P., Brečko B. N. (2013). DIGCOMP: A framework for developing and understanding digital competence in Europe.

[B29-behavsci-15-00366] Galindo-Domínguez H., Pegalajar M., Uriarte J.-D. (2020). Efecto mediador y moderador de la *resiliencia* entre la *autoeficacia* y el *burnout* entre el profesorado universitario de ciencias sociales y legales. Revista de Psicodidáctica.

[B30-behavsci-15-00366] Gao Y., Wang X., Reynolds B. L. (2025). The mediating roles of resilience and flow in linking basic psychological needs to tertiary EFL learners’ engagement in the informal digital learning of english: A mixed-methods study. Behavioral Sciences.

[B31-behavsci-15-00366] Glackin M., Hohenstein J. (2018). Teachers’ self-efficacy: Progressing qualitative analysis. International Journal of Research & Method in Education.

[B32-behavsci-15-00366] Guo X., Li X., Guo Y. (2024). Factors influencing the satisfaction of second language learners of chinese in online courses. Behavioral Sciences.

[B33-behavsci-15-00366] Hair J. F., Anderson R. E., Tatham R. L. (1987). Multivariate data analysis with readings.

[B34-behavsci-15-00366] Hammond M., Reynolds L., Ingram J. (2011). How and why do student teachers use ICT?. Journal of Computer Assisted Learning.

[B35-behavsci-15-00366] Han W. (2022). Chinese english as a foreign language teachers’ job satisfaction, resilience, and their psychological well-being. Frontiers in Psychology.

[B36-behavsci-15-00366] Hatlevik O. E. (2017). Examining the relationship between teachers’ self-efficacy, their digital competence, strategies to evaluate information, and use of ICT at school. Scandinavian Journal of Educational Research.

[B37-behavsci-15-00366] Hong A. J., Kim H. J. (2018). College students’ digital readiness for academic engagement (DRAE) scale: Scale development and validation. The Asia-Pacific Education Researcher.

[B38-behavsci-15-00366] Houlden S., Veletsianos G. (2020). Coronavirus pushes universities to switch to online classes–but are they ready. The Conversation.

[B39-behavsci-15-00366] Kibe C., Boniwell I. (2015). Teaching well-being and resilience in primary and secondary school. Positive Psychology in Practice: Promoting Human Flourishing in Work, Health, Education, and Everyday Life.

[B40-behavsci-15-00366] Krumsvik R. J. (2014). Teacher educators’ digital competence. Scandinavian Journal of Educational Research.

[B41-behavsci-15-00366] Kumar A., Kumar P., Palvia S. C. J., Verma S. (2017). Online education worldwide: Current status and emerging trends. Journal of Information Technology Case and Application Research.

[B42-behavsci-15-00366] Lan Y., Bin Saad M. R. (2024). Emotional presence in the community of inquiry: Addition of perma in online english teaching and learning. Asia-Pacific Education Researcher.

[B43-behavsci-15-00366] Lauridsen L. S., Willert M. V., Eskildsen A., Christiansen D. H. (2017). Cross-cultural adaptation and validation of the danish 10-item connor-davidson resilience scale among hospital staff. Scandinavian Journal of Public Health.

[B44-behavsci-15-00366] Levano-Francia L., Sanchez Diaz S., Guillén-Aparicio P., Tello-Cabello S., Herrera-Paico N., Collantes-Inga Z. (2019). Competencias digitales y educación. Propósitos y Representaciones.

[B45-behavsci-15-00366] Li Q., Gu Q., He W. (2019). Resilience of chinese teachers: Why perceived work conditions and relational trust matter. Measurement: Interdisciplinary Research and Perspectives.

[B46-behavsci-15-00366] Li S. (2023). The effect of teacher self-efficacy, teacher resilience, and emotion regulation on teacher burnout: A mediation model. Frontiers in Psychology.

[B47-behavsci-15-00366] Lin C.-H., Zheng B. (2015). Teaching practices and teacher perceptions in online world language courses. Journal of Online Learning Research.

[B48-behavsci-15-00366] Liu H., Chen B., Li X., Zhou X. (2024). Exploring the predictive role of self-efficacy in engagement among EFL teachers in online teaching: The mediation of buoyancy. The Asia-Pacific Education Researcher.

[B49-behavsci-15-00366] Liu H., Chu W. (2022). Exploring EFL teacher resilience in the Chinese context. System.

[B50-behavsci-15-00366] Liu H., Chu W., Wang Y. (2021). Unpacking EFL teacher self-efficacy in livestream teaching in the chinese context. Frontiers in Psychology.

[B51-behavsci-15-00366] Mackey A., Gass S. M. (2016). Second language research: Methodology and design.

[B52-behavsci-15-00366] Mansfield C. F. (2020). Cultivating teacher resilience: International approaches, applications and impact.

[B53-behavsci-15-00366] Mansfield C. F., Beltman S., Broadley T., Weatherby-Fell N. (2016). Building resilience in teacher education: An evidenced informed framework. Teaching And Teacher Education.

[B54-behavsci-15-00366] Means B., Toyama Y., Murphy R., Bakia M., Jones K. (2009). Evaluation of evidence-based practices in online learning: A meta-analysis and review of online learning studies.

[B55-behavsci-15-00366] Moorhouse B. L. (2023). Teachers’ professional digital competence after a period of online teaching: The case of Hong Kong primary school English-language teachers. Asia Pacific Education Review.

[B56-behavsci-15-00366] Namaziandost E., Heydarnejad T., Rahmani Doqaruni V., Azizi Z. (2023). Modeling the contributions of EFL university professors’ emotion regulation to self-efficacy, work engagement, and anger. Current Psychology.

[B57-behavsci-15-00366] Peng R., Hu Q. (2024). Measuring EFL teachers’ teaching anxiety in the online teaching environment. Asia Pacific Journal of Education.

[B58-behavsci-15-00366] Perera H. N., Calkins C., Part R. (2019). Teacher self-efficacy profiles: Determinants, outcomes, and generalizability across teaching level. Contemporary Educational Psychology.

[B59-behavsci-15-00366] Polin L. G. (2023). Self-efficacy and professional development of Chinese language teachers in North Carolina secondary schools. Language Teaching Research.

[B60-behavsci-15-00366] Rahimi A. R. (2024). Beyond digital competence and language teaching skills: The bi-level factors associated with EFL teachers’ 21st-century digital competence to cultivate 21st-century digital skills. Education and Information Technologies.

[B61-behavsci-15-00366] Romero-Tena R., Llorente-Cejudo C., Puig-Gutiérrez M., Barragán-Sánchez R. (2021). The Pandemic and changes in the self-perception of teacher digital competences of infant grade students: A cross sectional study. International Journal of Environmental Research and Public Health.

[B62-behavsci-15-00366] Sang G., Wang K., Li S., Xi J., Yang D. (2023). Effort expectancy mediate the relationship between instructors’ digital competence and their work engagement: Evidence from universities in China. Educational Technology Research and Development.

[B63-behavsci-15-00366] Schmid R. F., Borokhovski E., Bernard R. M., Pickup D. I., Abrami P. C. (2023). A meta-analysis of online learning, blended learning, the flipped classroom and classroom instruction for pre-service and in-service teachers. Computers and Education Open.

[B64-behavsci-15-00366] Shen D., Cho M.-H., Tsai C.-L., Marra R. (2013). Unpacking online learning experiences: Online learning self-efficacy and learning satisfaction. The Internet and Higher Education.

[B65-behavsci-15-00366] Stavraki C., Karagianni E. (2020). Exploring Greek EFL teachers’ resilience. Journal for the Psychology of Language Learning.

[B66-behavsci-15-00366] Sun P. P., Mei B. (2022). Modeling preservice Chinese-as-a-second/foreign-language teachers’ adoption of educational technology: A technology acceptance perspective. Computer Assisted Language Learning.

[B67-behavsci-15-00366] Tait M. (2008). Resilience as a contributor to novice teacher success, commitment, and retention. Teacher Education Quarterly.

[B68-behavsci-15-00366] Tschannen-Moran M., Hoy A. W. (2001). Teacher efficacy: Capturing an elusive construct. Teaching And Teacher Education.

[B69-behavsci-15-00366] Tschannen-Moran M., Hoy A. W., Hoy W. K. (1998). Teacher efficacy: Its meaning and measure. Review of Educational Research.

[B70-behavsci-15-00366] Wang M., Wang H., Shi Y. (2022). The role of English as a foreign language learners’ grit and foreign language anxiety in their willingness to communicate: Theoretical perspectives. Frontiers in Psychology.

[B71-behavsci-15-00366] Wang S., Wang Y., Zhao L. (2024a). Effects of psychological resilience on online learning performance and satisfaction among undergraduates: The mediating role of academic burnout. The Asia-Pacific Education Researcher.

[B72-behavsci-15-00366] Wang Y., Derakhshan A., Rahimpour H. (2024b). Developing resilience among Chinese and Iranian EFL teachers: A multi-dimensional cross-cultural study. Journal of Multilingual and Multicultural Development.

[B73-behavsci-15-00366] Wang Z., Chu Z. (2023). Examination of higher education teachers’ self-perception of digital competence, self-efficacy, and facilitating conditions: An empirical study in the context of China. Sustainability.

[B74-behavsci-15-00366] Xie F. (2021). A Study on Chinese EFL teachers’ work engagement: The predictability power of emotion regulation and teacher resilience. Frontiers in Psychology.

[B75-behavsci-15-00366] Xiyun S., Fathi J., Shirbagi N., Mohammaddokht F. (2022). A structural model of teacher self-efficacy, emotion regulation, and psychological wellbeing among English teachers. Frontiers in Psychology.

[B76-behavsci-15-00366] Yu X., Zhang J. (2007). Factor analysis and psychometric evaluation of the Connor-Davidson Resilience Scale (CD-RISC) with Chinese people. Social Behavior and Personality: An International Journal.

[B77-behavsci-15-00366] Zhang J., Xu F., Zhou Y., Wu J., Li Y., Qing W. (2024). Association between frailty and meaning in life of older adults in nursing home: The mediating effect of psychological resilience. Frontiers in Psychology.

[B78-behavsci-15-00366] Zhao Y., Pinto Llorente A. M., Sánchez Gómez M. C. (2021). Digital competence in higher education research: A systematic literature review. Computers & Education.

[B79-behavsci-15-00366] Zhi R., Derakhshan A. (2024). Modelling the interplay between resilience, emotion regulation and psychological well-being among Chinese English language teachers: The mediating role of self-efficacy beliefs. European Journal of Education.

